# Effects of paraprobiotic as replacements for antibiotic on performance, immunity, gut health and carcass characteristics in broiler chickens

**DOI:** 10.1038/s41598-022-27181-z

**Published:** 2022-12-31

**Authors:** Nampalle Mukesh Tukaram, Avishek Biswas, Chandra Deo, Agashe Jayanti Laxman, Madheshwaran Monika, Ashok Kumar Tiwari

**Affiliations:** Nutrition and Feed Technology Division, ICAR-Central Avian Research Institute, Izatnagar, Bareilly, Uttar Pradesh 243122 India

**Keywords:** Biochemistry, Microbiology

## Abstract

This study sought to determine the effects of dietary paraprobiotic (PPB) on broiler chicken performance, immunity, gut health, and carcass traits. A total of 240 day-old CARIBRO Vishal commercial broiler chicks of identical body weight randomly divided into six treatment groups, each with five replicates and eight chicks in each replicate. Six dietary treatments were preapared: T_1_ = (control diet), T_2_ = T_1_ + 0.02% (w/v) chlortetracycline (CTC), T_3_ = T_1_ + 0.2% (w/v) PPB, T_4_ = T_1_ + 0.4% (w/v) PPB, T_5_ = T_1_ + 0.6% (w/v) PPB and T_6_ = T_1_ + 0.8% (w/v) PPB, respectively. Body weight gain (BWG) significantly (P ≤ 0.05) increased in the T_5_ (0.6% PPB) and T_6_ (0.8% PPB) group. At the same time the feed intake significantly (P ≤ 0.05) decreased and the feed conversion ratio (FCR) significantly (P ≤ 0.05) improved in T_5_ and T_6_ group. There was a significant (P ≤ 0.05) increase in cell-mediated immunity and haem-agglutination titre (HA titre) in the 0.6% and 0.8% PPB supplemented groups compare to the control group (T_1_). The percentage of carcass traits and organ weights did not significantly differ between the PPB-supplemented and control groups, but the percentage of live weight in cut up parts showed a significant improvement (P ≤ 0.05) in the PPB-supplemented group. At 42 days, villus height, width, and crypt depth all significantly (P ≤ 0.05) increased in the groups supplemented with 0.6 and 0.8% para-probiotics (T_5_ and T_6_). The results show that para-probiotics can be added to broiler diets at a rate of 0.6% (w/v) to enhance performance, immunity, gut health, and breast yield. The para-probiotic may therefore be a useful substitution for antibiotic growth promoters in the diet of chickens.

## Introduction

One of the most productive agricultural enterprises is the poultry industry. The poultry industry's incredible growth can be attributed to sophisticated technologies developed as a result of research and progress in nutrition, genetics, housing management, chicken health and welfare. As a result, in recent decades, it has emerged as the fastest growing and most adaptable sector, with egg and broiler production growing at rates of 8.51 and 7.52%, respectively^1^. Antibiotics used as growth promoters have resulted in record levels of poultry production output worldwide. Antibiotics in animal feed have altered the intestinal flora of chickens and influenced their immunity, resulting in a greater ability to control disease^2,3^. On the other hand, unchecked and careless antibiotic use has led to the development of antibiotic resistant bacteria and an increase in the prevalence of antibiotic residues in animal products, threatening the health of both consumers and animals^4,5^. As a result, the majority of the European Union and other nations around the world forbid the use of antibiotics in animal feed. In order to find safer alternatives to antibiotics that have the same or better effects on animal production, there has been an increase in research^6^. Probiotics, prebiotics, symbiotics, and postbiotics have thus all recently been the focus of extensive research as antibiotic alternatives in poultry and livestock production.

Probiotics have many positive health effects, but their functionality and usefulness have come under scrutiny. Despite the fact that many probiotics have notable positive effects, mixed findings indicate that probiotics need to be more specifically tailored for animal species, and their general effectiveness is in question. Some probiotic bacteria have antibiotic-resistance genes that they can express and pick up from other microorganisms via plasmid transfer^7^. Additionally, probiotics were discovered to have a detrimental effect on the host by causing local inflammation in healthy hosts and escalating tissue inflammation in hosts with inflammatory bowel disease^8^. The current definition of a probiotic states that it must be alive; therefore, it does not apply to dead bacterial cells or cell parts. As a result, terms like paraprobiotic were developed to describe the health advantages beyond the probiotics' inherent viability, giving the probiotic concept a broad scope^9^. The food, biotechnology, and pharmaceutical industries are particularly interested in these terms because, despite their recent emergence, they have been adopted quickly in the fields of food science and technology, as well as in human health and nutrition, which has led to a special interest in their potential application as functional foods, nutraceuticals, and drugs^10^.

The use of non-viable probiotic preparations (paraprobiotics) has increased due to these worries and recent research showing that even non-viable microorganisms can benefit consumers in ways similar to their viable counterparts^11^. The use of non-viable microbes or microbial cell extracts has gained more attention due to the on-going safety concerns surrounding the consumption of live microorganism cells. These products may have a significantly longer shelf life while posing fewer health risks to consumers from microbial translocation and infection. Because they lower the risk of microbial translocation, infection, or heightened inflammatory responses, which have been linked to some probiotics, non-viable microbial cells may be safer than live probiotics^12^.

According to recent research, paraprobiotics provide health benefits to consumers through a variety of mechanisms, including immune system modulation (cell wall compounds may improve immune function), increased adhesion to intestinal cells (which inhibits pathogen growth), and secretion of various metabolites^11^. In probiotics-supplemented feed preparations, the ratio of viable to non-viable microorganisms may vary, and the population of dead cells may be higher than the population of viable cells^13^. The use of non-viable microbes or microbial cell extracts, which could significantly reduce shelf-life issues and eliminate the risks of microbial translocation and infection in the consumer, has attracted increased interest due to the ongoing safety concerns surrounding the consumption of live microorganism cells^14^.

Therefore, the objective of this study was to find out how the performance, immunity, gut health, and carcass characteristics of broiler chickens were affected by a para-probiotic (an inactive microorganism derived from *Lactobacillus acidophilus*).

## Materials and methods

### Statement of ethics

Following the guidelines of the "Committee for the Purpose of Control and Supervision of Experiments on Animals (CPCSEA) 2012" established under the "Prevention of Cruelty to Animals Act 1960" of the Indian Penal Code," the experimental procedures used in the study were approved by the Institutional Animal Ethics Committee (IAEC) (25 August 2019/Project No. 8). The study was carried out in compliance with the Animal Research: Reporting of in Vivo Experiments (ARRIVE) guidelines.

### Production of para-probiotic

The procedure described by Arai et al.^15^, with minor modifications, was used to prepare the para-probiotic. The *Lactobacillus acidophilus* was obtained from M/s Unique Biotech Pvt., Ltd, Hyderbad, Telangana, India and incubated in selective sterile media, namely MRS-broth, at 37 °C for 18–24 h. Then the bacterial culture was centrifuged at 6000 rpm for 10 min, followed by two washes in phosphate buffer solution (PBS) and germ-free purified water to collect free cells. Thermal inactivation was achieved by autoclaving the bacterial culture at 121 °C for 15 min while suspended in sterile distilled water (10^7^ cfu/ml). Culturing the resultant sample on nutrient-enriched media (De Man, Rogosa and Sharpe MRS) and confirmed inactivation as zero colony forming unit counts were observed during culture. The liquid inoculum was adjourned in germ-free purified water before being formed into a premix and added to feed at various levels. The main compositions of paraprobiotics are peptidoglycans, surface proteins, cell wall polysaccharides^16^.

### Management, diets, and experimental design of birds

An exclusively random design was used to conduct the experiment. A total of 30 replicate groups with 8 birds each were created using 240 day-old straight run (sex ratio 1) commercial broiler chickens (CARIBRO-VISHAL) of uniform body weight. CARIBRO-VISHAL chicks were obtained from experimental hatchery of the institute. Broiler chicken diets were used to create six different dietary treatments, namely T_1_ (control diet), T_2_ (T_1_ + 0.02% chlortetracycline − CTC), T_3_ (T_1_ + 0.2% w/v para-probiotic-PPB), T_4_ (T_1_ + 0.4% w/v PPB), T_5_ (T_1_ + 0.6% w/v PPB), and T_6_ (T_1_ + 0.8% w/v PPB). Table 1 lists the components and nutritional requirement of broiler chicken's basal diet. The 42-day feeding trial included providing the birds with ad libitum amounts of their respective feed and fresh water while also administering vaccinations in accordance with the standard vaccination schedule used at the institute's farm. The birds were given 24 h of light on the first day, then one hour less each day until they had 18 h of light, which they continued to receive until the trial's conclusion.

**Table 1:** Ingredient and chemical composition of basal feed.

Ingredients (g/kg diet)	Starter (0–3 weeks)	Finisher (4–6 weeks)
Maize	55.005	61.005
Soya bean	38.600	32.600
Rapeseed meal	3.000	3.000
Limestone	1.000	1.000
Di-Calcium phosphate	1.600	1.600
Salt	0.300	0.300
dl-Methionine	0.170	0.170
Lysine	0.010	0.010
TM Premix 1^a^	0.100	0.100
Vit Premix 2^b^	0.150	0.150
B Complex^c^	0.015	0.015
Choline chloride	0.050	0.050
**Chemical composition of basal diet**		
Crude protein (g/kg)	226	190.6
ME (MJ/kg)	11.85	12.25
Calcium (g/kg)	9.8	9.8
Available P (g/kg)	5.0	4.2
Lysine (g/kg)	12.8	10.4
Methionine (g/kg)	5.1	4.3

^a^TM premix (mg/kg) diet: MgSO_4_·5H_2_O-300; MnSO_4_·H_2_O-55; KI-0.4; FeSO_4_·7H_2_O-56; ZnSO_4_·7H_2_O-30; CuSO_4_·5H_2_O-4.

^b^Vitamin premix supplied per kg diet: vitamin A (retinol)-8250 IU; vitamin D3 (cholecalciferol)-1200 IU; vitamin- K (menadione)-1 mg.

^c^B complex supplied per kg diet: vitamin B1 (thiamine)-2 mg; Vit. B2, 4 mg; vitamin B2 (riboflavin)-10 µg; niacin (nicotinic acid)-60 mg; pantothenic acid-10 mg; choline, 500 mg.

### Production efficiency

Over the course of the experiment, weekly and overall body weight gains (BWG) measurements were made. Every day in the morning, a weighed quantity of each diet was given to each dietary regimen, and the residue was weighed the next day to determine the total amount of feed consumed. The weekly and period-wise feed conversion ratio (FCR) of birds was calculated using data from feed intake (FI) and BWG. The mortality of the experimental birds used in this study was investigated using daily observation and individual recording.

### Immune response

At the third week of experiment, broiler chickens were immunised (booster dose of *La-sota strain* vaccine through drinking water). Haem-agglutination (HA) test procedures were used to determine the antibody titre in a U-bottom micro titre plate^17,18^. Blood was collected from the jugular vein of healthy sheep using Alsever's solution. The blood was centrifuged at 2500 rpm for approximately 10 min. The red blood cells were thoroughly washed in phosphate buffer solution (PBS) three times before the supernatant was discarded. After washing, 100 ml of 1% sheep red blood cells (SRBC) suspension was created by combining 1 ml of SRBC with 99 ml of PBS. This suspension was then kept in the refrigerator at 4 °C until it was needed. To test the primary antibody response to SRBC, 24 birds per treatment received an intravenous injection of 1.0 ml of SRBC suspension on the 21st day after hatching. Two millilitres of blood were collected from the wing vein on the 26th day (5 days after vaccination). The serum was extracted after the blood had time to clot, and it was then frozen (at − 20 °C) until the antibody titres against SRBC could be determined. Before the haem-agglutination antibody (HA) titre was determined by a micro-haem agglutination method^19^ using two-fold serial dilutions of sera, the microtitre plate was first rinsed with 50 μl of PBS (pH 7.6) and added 50 μl of sera in the first well. Next, 50 μl of 1% SRBC in PBS was added in each well, and the wells were then dried.

Cell mediated immune (CMI) response was assessed in vivo by cutaneous basophilic hypersensitivity test using Phytohemagglutinin-P (PHA-P), according to Corrier and Deloach^19^. At 35 days, ten birds from each treatment were chosen, and the toe thickness of both the left and right foot at the third and fourth interdigital spaces was measured with a micrometre. Immediately following measurements, 100 mg of PHA-P suspended in 1 ml of phosphate buffer saline (PBS) and 0.2 ml of PBS were injected into the right and left feet, respectively (as a control). The web swelling in both feet was measured with a micrometre 24 h after injection, as described by Cheng and Lamont^20^. Foot Web Index was used to express the in vivo CMI response to PHA-P. Foot web swelling was calculated by subtracting skin thickness at 24 h post-injection from that at 0 h pre-injection.

### Carcass characteristics and cut up parts

At the end of the trial (42 days), four birds were chosen at random from each replicate of the treatment (20 birds per dietary treatment, n = 120) and sacrificed after 12 h of fasting with ad libitum drinking water for the assessment of carcass characteristics, organ weight, and cut up parts.

### Gut histomorphology

At 42 days of age, eight birds (n = 8) were euthanized and jejunum tissue samples were collected. On a glass slide, two cross-sections were prepared for each jejunum sample. A two cm section of the jejunum was removed, washed in physiological saline solution, and fixed in 10% buffered formalin. Following paraffin embedding of each segment, a two m section of each sample was placed on a glass slide and stained with hematoxylin and eosin for examination. An optical microscope (Motic Inverted microscope, Honkong), a camera (Motic cam, CMOS, Honkong), and image analysis software were used to measure all jejunum sample (Motic Image 2.0, Honkong). In each segment evaluated, the morphometric indices used were villus height, villus width, crypt depth, and the villus height to crypt depth ratio. The average jejunum of each bird was calculated. The ratio of villus height to crypt depth was then calculated.

### Statistical analysis

The bird that was sampled served as the experimental unit for data analysis. After being completely randomised, the data were analysed using one way ANOVA and the General Linear Model procedure (IBM SPSS softeware-20). Tukey post-hoc analysis was used to test for significant mean differences between groups, with P ≤ 0.05 set as the level of significance.

## Results

### Production performance

Table 2 displays the results of the growth performance. In comparison to treatments T_1_ and T_2_, the body weight gain (BWG) of chickens increased significantly (P ≤ 0.05) during the 4–6 and 0–6 week time periods, and feed intake (FI) decreased significantly (P ≤ 0.05) in T_5_ and T_6_ group. The result also showed that treatments T_5_ and T_6_ significantly (P ≤ 0.05) improved feed conversion ratio (FCR) between 0 and 6 weeks of age in comparison to T_1_ and T_2_.

**Table 2:** Effect of para-probiotic on production performance in broiler chickens (0–6 weeks).

Parameters	Dietary treatment						SEM	P-value
	T1	T2	T3	T4	T5	T6		
**Body weight gain (g)**								
0–3 weeks	554.73	565.34	570.82	571.11	583.27	585.10	6.22	0.235
4–6 weeks	1055.27^a^	1064.31^a^	1052.82^a^	1053.75^a^	1116.03^b^	1111.16^b^	12.67	0.042
0–6 weeks	1521.03^a^	1530.62^a,b^	1523.62^a^	1545.85^a,b^	1652.25^b^	1625.12^b^	6.52	0.023
**Cumulative feed intake (g)**								
0–3 weeks	982.20	938.81	960.16	972.87	905.62	915.15	15.02	0.412
4–6 weeks	2260.42	2192.80	2132.63	2221.65	2170.21	2188.86	28.47	0.065
0–6 weeks	3033.20^b^	3021.61^b^	2992.24^b^	2974.45^b^	2884.80^a^	2891.22^a^	35.62	0.011
**Feed conversion ratio (FCR)**								
0–3 weeks	1.77^b^	1.66^a,b^	1.68^a,b^	1.7^b^	1.55^a^	1.56^a^	0.16	0.010
4–6 weeks	2.14	2.06	2.03	2.11	1.94	1.97	0.21	0.242
0–6 weeks	1.99^b^	1.97^b^	1.96^b^	1.92^ab^	1.75^a^	1.78^a^	0.14	0.001

Mean values bearing the same superscript in a row did not differ significantly (P < 0.05).

T_1_ = control, T_2_ = 0.02% CTC (w/v), T_3_ = 0.2% PPB (w/v); T_4_ = 0.4% PPB (w/v); T_5_ = 0.6% PPB (w/v); T_6_ = 0.8% PPB (w/v).

*CTC* Chlortetracycline.

### Immune response

The dietary addition of para-probiotic at 0.6% (T_5_) had a significant impact on the immune response of birds and the weight of immune organs (Table 3). Treatment T_5_ had the highest (P ≤ 0.01) index of humoral immunity and cell-mediated immunity, followed by T_6_, T_4_, and T_1_. The results of the study showed that higher para-probiotic incorporation treatment had a significant impact on the weight of immune-related organs like the spleen (P ≤ 0.01) and thymus (P ≤ 0.05) (T_5_ and T_6_).

**Table 3:** Effect of dietary para-probiotic on immune response and relative immune organ (%) in broiler chickens.

Parameter	Dietary treatment						SEM	P-value
	T1	T2	T3	T4	T5	T6		
**Immune response**								
Humoral (log_2_)	1.79^a^	2.40^b^	1.90^a^	2.50^b^	2.84^b^	2.68^b^	0.092	0.006
Cell mediated (mm)	0.67^a^	0.93^b^	0.70^a^	0.85^a,b^	0.99^b^	0.91^b^	0.062	0.000
**Related Immune organ (%)**								
Spleen	0.18^a^	0.23^a,b^	0.21^a,b^	0.22^a,b^	0.25^b^	0.24^a,b^	0.03	0.005
Bursa	0.16	0.17	0.15	0.17	0.18	0.17	0.01	0.075
Thymus	0.32^a^	0.32^a^	0.30^a^	0.34^a,b^	0.36^b^	0.35^b^	0.04	0.035

Mean values bearing the same superscript in a row did not differ significantly (P < 0.05).

T_1_ = control, T_2_ = 0.02% CTC (w/v), T_3_ = 0.2% PPB (w/v); T_4_ = 0.4% PPB (w/v); T_5_ = 0.6% PPB (w/v); T_6_ = 0.8% PPB (w/v).

*CTC* Chlortetracycline.

### Carcass characteristic and organ weight

Tables 4 and 5 show the results for organ weight and carcass characteristics. Between all dietary treatments, there was no discernible difference in carcass characteristics or different organ weights. According to the study, T_6_ had significantly (P ≤ 0.05) higher yields of thigh, breast, and drumstick weights than T_5_, T_4_, T_2_, or T_1_. However, T_1_ and T_3_ were statistically similar.

**Table 4:** Effect of dietary para-probiotic on carcass traits and organ weight (% of live weight) in broiler chickens.

Parameter	Dietary treatment						SEM	P-value
	T1	T2	T3	T4	T5	T6		
**Carcass traits (%)**								
Feather loss	5.79	5.67	5.90	4.90	5.48	5.68	0.09	0.076
Dressing yield	63.22	62.54	64.10	63.82	64.75	65.15	0.66	0.102
Eviscerated yield	69.10	69.45	71.25	69.90	71.12	72.02	1.72	0.061
**Organs weight**								
Abdominal fat	0.92	1.06	1.01	0.88	0.93	0.94	0.003	0.085
Heart	0.66	0.65	0.65	0.66	0.62	0.64	0.02	0.121
Liver	2.62	2.45	2.52	2.60	2.62	2.59	0.12	0.202
Gizzard	2.04	2.10	2.25	2.36	2.42	2.40	0.16	0.072

Mean values bearing the same superscript in a row did not differ significantly (P < 0.05).

T_1_ = control, T_2_ = 0.02% CTC (w/v), T_3_ = 0.2% PPB (w/v); T_4_ = 0.4% PPB (w/v); T_5_ = 0.6% PPB (w/v); T_6_ = 0.8% PPB (w/v).

*CTC* Chlortetracycline.

**Table 5:** Effect of dietary para-probiotic on cut up parts weight (% of live weight) in broiler chickens.

Parameter	Dietary treatment						SEM	P-value
	T1	T2	T3	T4	T5	T6		
Thigh	9.75^a^	10.06^ab^	9.65^a^	9.97^a,b^	10.36^b^	10.55^b^	1.12	0.032
Back	18.24	18.45	19.05	18.88	19.20	19.45	3.56	0.132
Drumstick	10.26^a,b^	10.28^a,b^	9.86^a^	10.32^a,b^	10.65^b^	10.75^b^	1.47	0.002
Breast	15.63^a^	16.20^a^	16.43^a,b^	16.68^a,b^	17.07^b^	17.18^b^	2.15	0.000
Wings	8.03	8.10	7.99	8.12	8.16	8.20	0.97	0.111
Neck	3.25	3.45	3.67	3.66	3.65	3.70	0.23	0.062
M:B (Thigh)	5.10	5.20	4.85	4.92	5.34	5.22	0.78	0.082
M:B (Breast)	6.11	5.78	5.87	5.90	6.25	6.15	1.06	0.105

Mean values bearing the same superscript in a row did not differ significantly (P < 0.05).

T_1_ = control, T_2_ = 0.02% CTC (w/v), T_3_ = 0.2% PPB (w/v); T_4_ = 0.4% PPB (w/v); T_5_ = 0.6% PPB (w/v); T_6_ = 0.8% PPB (w/v).

*CTC* Chlortetracycline, *M:B* Meat bone ratio.

### Gut morphology

The para-probiotic supplemented groups, T_6_ (0.8% PPB w/v) and T_5_ (0.6% PPB w/v), had significantly higher villus height (VH), width (VW), crypt depth (CD) at 42 days compared to other treated groups (Table 6). There were no discernible variations in the ratio of villus height to crypt depth (VH: CD).

**Table 6:** Effect of dietary para-probiotic on intestinal histo-morphometry in broiler chickens.

Parameter	Dietary treatment					SEM		P-value
	T1	T2	T3	T4	T5	T6		
Villus width (µm)	110.28^a^	118.78^a^	123.16^a^	129.25^a,b^	146.20^b^	145.55^b^	5.23	0.024
Villus height (µm)	1086.1^a^	1089.7^a^	1156.80^a,b^	1245.19^a,b^	1290.40^b^	1280.22^b^	18.7	0.019
Crypt depth (µm)	190.8^a^	187.2^a^	207.31^a,b^	212.0^b^	225.22^b^	220.10^b^	7.90	0.034
VH:CD	5.69	5.82	5.58	5.87	5.73	5.82	0.72	0.069

Mean values bearing the same superscript in a row did not differ significantly (P < 0.05).

T_1_ = control, T_2_ = 0.02% CTC (w/v), T_3_ = 0.2% PPB (w/v); T_4_ = 0.4% PPB (w/v); T_5_ = 0.6% PPB (w/v); T_6_ = 0.8% PPB (w/v).

*CTC* Chlortetracycline.

## Discussion

This study found that, when compared to the control, antibiotic-treated group, diets containing paraprobiotics had an impact on growth performance at 4–6 and 0–6 weeks of age. The performance of the chickens given paraprobiotic supplements was on par with, and in some cases even better than, that of the positive control group (chickens fed antibiotics). This might be as a result of the paraprobiotic's bacteriostatic and bactericidal qualities, which are responsible of lowering pathogenic bacteria in the gut. They may therefore function similarly to antibiotics in terms of enhancing growth performance. According to the results of the current study, paraprobiotics had antibacterial activity against pathogenic bacteria. In order to prevent the growth of harmful bacteria, paraprobiotics naturally contain antibacterial substances. This discovery further demonstrated that paraprobiotics, like antibiotics, can inhibit the growth of harmful bacteria while reducing their numbers. They are therefore suitable alternatives to antibiotics. Due to their capacity to enhance livestock performance, probiotics have recently drawn considerable attention and are establishing themselves as a secure and practical substitute for antibiotics^21^. Contrarily, because they include functional fermentation substances like short-chain fatty acids, microbial fractions, functional proteins, secreted polysaccharides, extracellular polysaccharides, cell lysates, teichoic acid, peptidoglycan-derived muropeptides, and pili-type structure^22,23^ and can be combined with other substances to enhance animal health, paraprobiotics are safer than probiotics^24^.

However, between 0 and 6 weeks, the FCR significantly improved. When contrasted with the control and other treated groups, the T_5_ and T_6_ groups' birds had the lowest FCR values. The results of Danladi et al.^25^ are at odds with this because they discovered no appreciable modifications to broiler diets following the addition of paraprobiotics to the diet. The results of this study corroborate those of Humam et al.^26^, who reported improved FCR after dietary supplementation with paraprobiotic and postbiotic bacteria. Many variables affect the health and performance of birds, including environmental stress, diet management, farm sanitation, unidentified microorganisms, and bird age^27^. But the present study discovered that dietary paraprobiotic supplementation significantly enhanced bird growth performance at age’s 0–3 and 0–6 weeks.

These results are in line with those of Xiao et al.^28^ and Hand^29^, who discovered that the anti-vaccine titre of birds that had been given killed probiotic treatment was significantly higher than that of control birds. On the other hand, according to Donaldi et al.^25^, there were no appreciable differences in immune response when paraprobiotic or postbiotic supplementation was combined with a basal diet. According to this study, intake paraprobiotics significantly influenced immune system related organs like the spleen and thymus. In addition to acting as an agent and attaching to bacteria to initiate immune response, dietary paraprobiotics may also directly promote the immune system by active groups and compete with pathogens for nutrients, as well as inhibit specific pathogen colonisation in bird guts. These pathogens are, however, allowed to present themselves to immune cells as attenuated antigens. The immune response in chicken fed diets supplemented with para-probiotics is stronger, as evidenced by an increase in diffused lympho-histiocytic infiltration and solitary lymphoid follicles in the mucosa^30^. B cells are part of the immune system and are responsible for producing immunoglobulins (Ig), which are crucial for immune control and mucosal defence^31^. However, environmental stressors can affect the development of B cells^26^. IgA is crucial for defending mucosal surfaces by preventing pathogens and toxins from attaching to, colonising, and entering those^26^. Probiotics in animal feed can stimulate the immune system by migrating through the intestinal wall as viable cells and multiplying to a limited extent, causing the production of immunogenic compounds and mediating the down-regulation of specific signalling pathways^32^. Following that, increased macrophage activity and a systemic antibody response via increased production of immuneglobulins (IgG, IgM), interferon, IgA levels at mucosal surfaces, and the expression of various pro and anti-inflammatory cytokines may occur^33,34^.

According to the current study, there were no significant differences between treatments for carcass characteristics like eviscerated yield, dressing yield and weight of the heart, liver, and gizzard. The effects of paraprobiotics on the carcass yield of broiler chickens were not significantly different. Similar results were obtained by Humam et al.^26^, who found that para-probiotics fed to broiler chickens had no impact on carcass yield. Similar to this, when para-probiotic and inulin were fed to broiler chickens, the carcass yield was unaffected^35^. With regard to cut of parts yields, paraprobiotic treatment increased thigh, neck, breast, and drumstick yields between control and treated groups, with the exception of the back and wings (%), which were comparable^36^. On the other hand, Pelicano et al.^37^ discovered no variations in the yield of cut up parts between control and probiotic treated birds. This study did not discover any significant effects on abdominal fat. The paraprobiotic groups displayed a decrease in abdominal fat when compared to the control. This was in line with the findings of the study by Loh^38^, which showed that PPB may be useful in reducing the problems caused by high cholesterol levels in meat. Moreover, it has been demonstrated that the *Lactobaccilus acidophilus* bacterium reduces fat deposits in chickens^24^. In this study, some organ weights, including those of the heart, liver, and gizzard, were measured, but no differences in the weights of the non-carcass components were discovered. However, no treatment was found to have a significant impact on the overall characteristics of the bird carcass or the weight of its organs.

According to the results of the current study, PPB has a more consistent impact on the microstructure of the gut than control diets. They have an impact on villus height, width and crypt depth in the jejunum. This suggests that compared to control treatments, these chickens' jejunums had a higher level of absorptive function. Measurements of intestinal morphology that indicate increased nutrient absorption include increased villus height, short crypt depth, and other measures^24^. Additionally, villus height and crypt depth play a crucial role in animal health and digestion^35^. This result is consistent with a recent study by Humam et al.^26^ on probiotics and improved histo-morphology. According to Jha et al.^24^, longer villi signify increased feed efficiency and growth promoting efficiency. Another noteworthy discovery was a rise in villi heights, VH: CD ratios, and a decrease in crypt depths when compared to the control groups, which was consistent with the findings of the Humam et al.^26^.

The results of the current study are in agreement with those of Iji et al.^39^, who found that chickens fed control diets had significantly longer jejunum villi. The intestine can alter its surface area by lengthening and/or altering the height of its villi when paraprobiotics are consumed. Longer villi and shallower crypts have the opposite effect from shortening and fusion, which reduces the surface area available for food digestion and absorption^40,41^. It is well known that dietary probiotics alter the gut microflora significantly, frequently favouring the host, and that the gastrointestinal tract (GIT) is able to adapt and change its morphology in response to changing conditions like a changed diet^41^. The expected detrimental effects of nitrogenous substances on villus height were therefore monitored by measuring the jejunum histology. Villi are structures that function as areas where nutrients can be absorbed, so shorter villi indicate a decrease in the surface area for nutrient absorption from the gut^42^. Height enhanced the transport of nutrients across the villus surface^43^. The fact that broilers fed antibiotics have shorter villi and deeper crypts may indicate that the harmful substances created by microbial fermentation have caused more damage to the gut. The need for more protein and energy to accomplish this task was indicated by a deeper crypt, which also indicated increased enterocyte turnover. Crypt depth represents the amount of crypt cells generated^44^.

## Conclusions

According to the findings of this study, broler chicken performance, immunity, and gut morphometry were all improved when para-probiotics (PPB) were present at 0.6% (w/v). However, in the birds that received more paraprobiotic, the yield of some cut-up parts was higher. This study also shows that the para-probiotic has the potential to replace antibiotics in broiler chicken diets, but further study is required.

## Data Availability

Data will be available from the corresponding author upon a reasonable request. The experimental procedures on the birds were carried out in accordance with the ethical standards laid down in the 1964 Declaration of Helsinki and its later amendments. The experimental procedures and animal welfare measures were approved by the Institutional Animal Ethics Committee (IAEC) of Central Avian Research Institute, Izatnagar (IAEC approval number: 25 August 2019/Project No. 8).
